# Antenatal psychosomatic programming to reduce postpartum depression risk and improve childbirth outcomes: a randomized controlled trial in Spain and France

**DOI:** 10.1186/1471-2393-14-22

**Published:** 2014-01-15

**Authors:** Maria Assumpta Ortiz Collado, Marc Saez, Jérôme Favrod, Marie Hatem

**Affiliations:** 1 La Source, School of Nursing Sciences, University of Applied Sciences of Western Switzerland, 30 Avenue Vinet, CH-1004 Lausanne, Switzerland; 2 Department of Statistics Appliqued in Medicine, Research Group on Statistics, Econometrics and Health (GRECS), University of Girona, Ave. Montilivi s/n, 17071 Girona, Spain; 3 Social and Preventive Medicine Department, University of Montreal, 7101 Avenue du Parc, Montreal QC H3N 1X7 Canada

**Keywords:** Postpartum depression, Psychosomatic approach, Pregnancy, Antenatal group preparation, Childbirth outcomes

## Abstract

**Background:**

Postpartum depression (PPD) and poor childbirth outcomes are associated with poverty; these variables should be addressed by an adapted approach. The aim of this research was to evaluate the impact of an antenatal programme based on a novel psychosomatic approach to pregnancy and delivery, regarding the risk of PPD and childbirth outcomes in disadvantaged women.

**Methods:**

A multi-centre, randomized, controlled trial comparing a novel to standard antenatal programme. Primary outcome was depressive symptoms (using EPDS) and secondary outcome was preterm childbirth (fewer 37 weeks). The sample comprised 184 couples in which the women were identified to be at PPD risk by validated interview. The study was conducted in three public hospitals with comparable standards of perinatal care. Women were randomly distributed in to an experimental group (EG) or a control group (CG), and evaluated twice: during pregnancy (T1) and four weeks post-partum (T2). At T2, the variables were compared using the chi square test. Data analysis was based on intention to treat. The novel programme used the Tourné psychosomatic approach focusing on body awareness sensations, construction of an individualized childbirth model, and attachment. The 10 group antenatal sessions each lasted two hours, with one telephone conversation between sessions. In the control group, the participants choose the standard model of antenatal education, i.e., 8 to 10 two-hour sessions focused on childbirth by obstetrical prophylaxis.

**Results:**

A difference of 11.2% was noted in postpartum percentages of PPD risk (EPDS ≥ 12): 34.3% (24) in EG and 45.5% (27) in CG (p = 0.26). The number of depressive symptoms among EG women decreased at T_2_ (intragroup p = 0.01). Premature childbirth was four times less in EG women: three (4.4%) compared to 13 (22.4%) among CG women (p = 0.003). Birth weight was higher in EG women (p = 0.01).

**Conclusions:**

The decrease of depressive symptoms in women was not conclusive. However, because birth weight was higher and the rate of preterm childbirth was lower in the EG, our results suggest that the psychosomatic approach may be more helpful to the target population than the standard antenatal programs.

## Background

Between 10 and 15% of new mothers show depressive symptoms [1,2]. This prevalence justifies prevention of this universal public health problem [3], particularly in women with low socioeconomic status, of whom nearly one in four suffers from postpartum depression (PPD) [4,5]. Although early PPD detection is effective [6], in many countries – including Spain and France – there are few protocols in place. Furthermore, it is important to avoid chronicity, as many cases of untreated PPD continue and at times worsen during this period [7].

In the *Diagnostic and Statistical Manual of Mental Disorders *[8], PPD is categorized as a major depression and is defined as a depressive episode appearing within four weeks after childbirth. Some authors describe it as a medical/psychiatric disorder that includes both psychological and physiological aspects [9]. Others, inspired by the sociological model, postulate that certain mothers become vulnerable due to a lack of social support combined with the presence of negative social factors [10,11]. It is important to differentiate between non-psychotic depression and two other postnatal affective disorders: baby blues and postpartum psychosis.

Non-psychotic PPD involves a great feeling of guilt about finding the maternal role difficult to assume, desperation, sleeping disorders, depressive mood, anxiety, loss of concentration, negative thoughts of oneself, and even thoughts of death [8]. However, the sadness that characterizes depression is not the main sign in many depressed mothers [12]. Less obvious symptoms, such as certain somatization disorders, may hide the problem and, in the end, neither the women themselves nor professionals can identify the source of the health problem. The woman feels isolated and is unable to understand what is happening to her while no one diagnoses or treats her [13,14]; this failure to recognize the problem can lead professionals to interpret symptoms erroneously [15]. According to Chee et al. [16], when a mother does not realize what is happening to her, she may overlook some aspects of her baby’s health. Her PPD affects all members of the family [17], particularly the baby. The foetus may be influenced by the mother’s antenatal depression; this translates into elevated foetal activity and a high frequency of underweight and premature births among depressed mothers [18-21]. Moreover, the temperament of these babies is different from that of babies born to non-depressed mothers; they display more difficult behaviour [22].

Risk factors very closely associated with PPD are poverty, absence of a partner or separation during pregnancy, and/or unwanted pregnancy [23]. To these, one may add prenatal depression, lack of social support, anxiety, conflict with the partner [24] and physical or psychological violence in intimate partner relationships [25,26]; a number of these psychosocial factors may influence preterm birth.

Some authors also associate PPD with depression and anxiety during pregnancy, stressful events before and after the birth, unemployment, and recent immigration [27]. At least one stressful situation was reported by 41% of women with PPD studied by Yelland et al. [28], such as high economic difficulties, job loss, separation or divorce.

Other authors have stated that if the level of prenatal stress is reduced in depressed women, the indicators of depression also tend to decrease [20,29]. Vieten and Astin [30] report that preventive intervention using mind control had a positive impact on the anxiety and negative feelings experienced by pregnant women in an experimental group. Although the low number of participants – 13 in the experimental group and 18 in the control group – did not enable a generalization of the results, the study did open a path for further research. Prior studies had demonstrated the favourable impact of stress reduction techniques – such as massage – on the depressive mood of pregnant women; this also benefited their babies by significantly reducing the rate of premature births (0% EG vs. 17% CG) and improving the behavioral responses of babies on the Brazelton scale [20,31]. Applying interpersonal psychotherapy to 99 pregnant women from low socioeconomic backgrounds, the authors found significantly fewer women at risk of PPD after intervention [32]. A psycho-educational intervention that targeted 377 pregnant women reduced PPD risk in EG participants with high initial depressive symptoms and was evaluated by the *Beck Depression Inventory* (BDI)-II [33]. Other preventive programmes using pharmacological intervention found no differences in PPD between EG and CG women [34,35]. Although constant progress exists in methods for PPD prevention, thus far only interpersonal therapy, psycho-educational intervention and massage practice were effective. Massage practice has proven effective in reducing two associated variables: PPD and preterm childbirth [20].

Most of these PPD prevention studies were conducted in the United States and in Australia but rarely in Europe and never in Southern Europe. The intervention strategies included cognitive behavioral therapy [36-39], interpersonal psychotherapy [32,40-43], and psycho-educational intervention [33,44-47], but never any psychosomatic approach. These studies did not include preterm birth variables in outcome evaluations. Prevention of PPD is justifiable, however a special approach would be advisable in a group of women who are at high risk for adverse perinatal outcomes, such as PPD, preterm birth, and low SES, because a complex group of women requires a complex psychosomatic intervention.

Mental health and mother-baby health are priorities of national health plans. Hence, the psychosomatic intervention method presents a novel approach to antenatal intervention.

The preterm childbirth (fewer 37 weeks) rate is difficult to reduce and to date remains unchanged despite prevention efforts: between 5 and 8% in Europe [48] 13% and 15% in the United States [49]. The proposed psychosomatic approach takes a different view of depressive symptoms and is related to the understanding of certain physical symptoms and awareness of new sensations during pregnancy that can influence premature childbirth. Some authors suggest that PPD prevention should take place when women visit health centres during pregnancy, as it is easy to detect depressive or somatic symptoms indicative of PPD risk [14]. Prenatal symptoms can be detected using a psychosomatic approach related to life situations. Raising awareness and understanding of the different symptoms can reduce stress levels among pregnant women, which in turn improve childbirth outcomes such as preterm birth [20].

The hypothesis of the present study is that participants in the antenatal programme, based on a psychosomatic approach and evaluated using the Edinburgh Postnatal Depression Scale (EPDS ≥12) four weeks after childbirth, would present at least 6% fewer cases of PPD risk than women who did not participate in the programme. A cut-off score of ≥12 is recommended in Spanish studies when assessing the risk of PPD [50]. The 6% difference is calculated in the event that the prevalence of PPD is 13% as indicated by meta-analysis of O’Hara and Swain [1]. The percentage of preterm birth is between 5 and 15%, so the 13% used in this study for sample size estimation would be appropriate to both PPD and preterm birth.

The overall aim of this study was to evaluate the impact of a novel psychosomatic antenatal programme meant to decrease symptoms of depression (primary outcome) evaluated using the EPDS in the fourth week after childbirth, and preterm birth (secondary outcome). The study included two groups of women (CG and EG) identified in the prenatal period as being at risk of PPD with regard to validated interview.

## Methods

This was a multicentre randomized, longitudinal clinical study using intention to treat in data analysis. Pregnant women at risk of PPD (evaluated by validated interview) and their partners were randomly assigned to a control group (CG) or an experimental group (EG) by a random sampling allocation sequence. The allocation to the study groups was blinded; all interviews were sent to an outside statistician who never met the participants. The statistician telephoned the researcher to notify the assignment of eligible women to control groups or experimental groups. A second statistician evaluated the other questionnaires. Participants knew they were in a study group but did not know about the distinction between control and experimental intervention. The nurse midwives who ran the control group also had no prior knowledge. Only nurse midwives who animated the experimental group knew about the distinction but never had access to the questionnaires and never knew the evaluated variables.

### Setting

Two assessments were made. The initial evaluation (T_1_) took place in the hospital during pregnancy when women came for the first or second echography. The women were asked to complete a questionnaire. The follow-up evaluation (T_2_) questionnaire was sent by mail at one month postpartum, to be self-administered and returned before the 12^th^ week after childbirth (Table 1). The study was conducted progressively in three cities: Barcelona, Figueres and Beziers from May 2003 through July 2007 with final analyses in 2008 and additional analyses in 2009 (Table 2).

**Table 1:** Testing variables

**Variables**	**Evaluation instruments**	**Quality of instruments**	**Time application instruments**
Demographic & obstetric data	Included in antenatal interview		Antenatal
Antenatal risk of PPD	**Interview** (Riguetti-Veltema & et. al, [14])	Sensitivity = 84,5%, Specificity = 73%	Antenatal
Symptoms of depression	EPDS*Edinburgh Postnatal Depression Scale (Cox & et. al, [51])	Sensitivity = 86%, Specificity = 78%	Antenatal and postnatal
Relationship with partner	DASS*Dyadic Adjustement Scale (Spanier, [52])	Fidelity: *Cronbach Alpha* = 0.96	Antenatal and postnatal
Social support	FSSQ*Functional Social Support Questionnaire (Broadhead, [53])	Fidelity: *Cronbach Alpha = 0.90*	Antenatal and postnatal
Stress	Stress events* (Holmes & Rahe, [54])		Antenatal and postnatal
Birth outcomes	**Hospital clinical data** and post-partum questionnaire*		Postnatal

*self-administered.

**Table 2:** Participants according to the different centres

**Centres**	**Participants**	**Groups**
Barcelona maternity hospital	260 interviewees	5
Figueres hospital	199 interviewees	3
Beziers hospital	70 interviewees	1
Total	529 interviewees	9

### Group leaders

Three nurse-midwives, one from each of the three public hospital participants, were the group leaders and Tourné, the intervention’s designer, conducted one group intervention in each centre. One nurse-midwife in each hospital volunteered to participate and received specific training (20 h), supervision and additional feedback.

### Participants

The participants were pregnant women and their partners considered to be at psychosocial risk via three factors: socioeconomic status (low-paid jobs, unemployed, with or without subsidy), low social support (migrants or those living isolated), and the risk of PPD (validated interview). Couples were eligible to take part in the study if (1) they were identified at middle or low socio economic status (based on income, occupational category and type of employment contract, an indicator of job security), and (2) the women met these individual criteria: a) pregnancy ≤ 20 weeks; b) a moderate to high risk of PPD; c) no more than two children; d) no organic serious physical pathology; e) no psychiatric diagnosis; f) no alcohol or illicit substance abuse, and (g) understand the language of the study.

Exclusion criteria included having a current diagnosis of psychiatric disorder or a serious medical condition. The participating couples signed consent forms and completed a questionnaire about their relationship; women completed all other questionnaires. The selection interview was used to assess the initial risk of PPD in women. This antenatal information was sent to the statistician who randomized the eligible couples to the control or experimental group using the SPSS.15 software.

**Figure 1:**
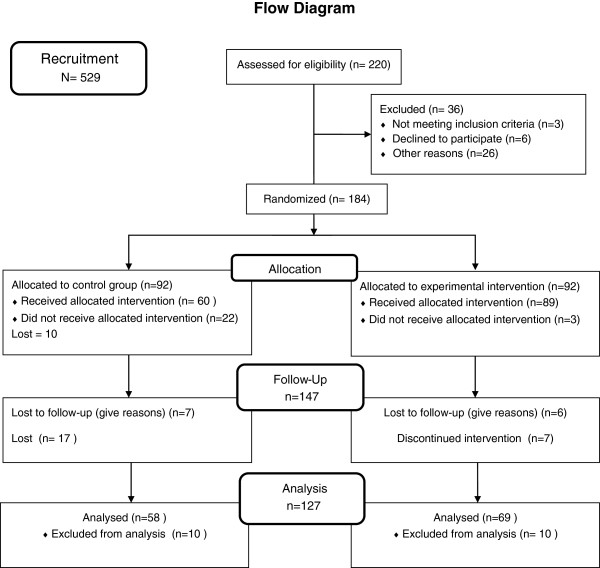
Consort diagram outlining study recruitment and retention.

The Righetti-Veltema et al. [14] antenatal interview with multicentre validation was used to determine the antenatal risk of PPD in women in the selection process. To this end, 529 women were interviewed but only 220 at risk of PPD were selected as eligible, 302 women (57.09%) were rejected for currently not being at risk of PPD. In addition, 36 couples were excluded from the data analysis for presenting a risk for bias or for refusing to continue in study. The final sample was composed of 184 couples. A statistician produced a computer-generated random distribution of women with antenatal risk of PPD in both groups, EG and CG (Figure 1). Another researcher with no prior knowledge of the participant data conducted the final data analysis.

### Sample size estimation

The sample size was estimated according to PPD prevalence rates [1] and premature childbirth prevalence rates [48,49]. Assuming a rate of 13%, a group difference of half that rate (6.5%) was selected as a reference. The group difference is based on a sample of 200 subjects, with the significance level set at 5% risk of failure (= 0.05), and the statistical power is 94%. In a sample of 150 subjects, the power obtained is 88%. Although the ideal sample size would have been 200 couples, the limited material resources available for the intervention meant that the study was conducted with fewer participants.

### Outcome measures

Data were collected by using a set of four self-administered questionnaires before intervention and again as follow-up questionnaires mailed between five and 12 weeks postpartum. A phone call invited the couples to complete the self-administered follow-up questionnaires. A reminder phone call was made, if necessary. The Righetti-Veltema et al. interview [14], used only for participant selection, consists of 14 questions related to the mother’s personal, social, psychological and physical situation. These include age, education, country of origin, pregnancy and childbirth history, relationship with own mother, intention to receive antenatal education, perception of the pregnancy, and seven questions relating to somatic symptoms; this is a novelty, because somatic symptoms do not appear in other instruments for the early detection of postnatal depression. This interview was validated in three countries: France, Spain and Switzerland but still not new articles have been published studies using this instrument. The implementation and evaluation of this interview is easy; for each question, there are four possible answers and these are scored. Questions on issues such as relationship with the mother, the partner, and frequent crying, were cross evaluated. The PPD risk in this instrument may range from 0 (no risk) to 10 (high risk); a score ≥ 3 indicates risk.

Four variables associated with PPD were assessed: 1) the depressive symptoms, using the Cox et al. 1987 [51] EPDS scale; 2) amount of social support received, using the *Functional Social Support Questionnaire* of Broadhead et al. 1988 [53]; 3) stressful events, based on Holmes and Rahe, 1967 [54]; and 4) relationship with the partner using Spanier’s dyadic adjustment scale *DAS,* 1976 [52], applied separately for women and men. For the evaluation of these variables from self-reported data, a questionnaire was made for all postnatal variables applying to the mother’s health, postpartum somatic symptoms, and childbirth (Table 1). For better reliability, preterm childbirth (considered as < 37 weeks) and baby weight was verified with the clinical history.

### Ethical approval

The research centre at the Barcelona Clinic Hospital approved this study (File Record 828). This study conformed to procedures in accordance with ethical standards on human experimentation.

### Interventions

The experimental programme took a psychosomatic approach based on a humanist intervention theory that develops awareness of feelings and body sensations, their differentiation and their interrelationship. It was conceived by Tourne [55] and uses humanistic and cognitive techniques such as: developing a therapeutic alliance based on the participant’s perspective, normalizing antenatal somatic symptoms, developing alternative explanations for their sensations and experience, and connecting somatic symptoms to emotion. Each session has two or more specific objectives which are worked toward progressive stages, as well as exercises for reasoning with somatic symptoms and childbirth model; sessions number five and seven are open and without topic, and serve to answer questions and clarify doubts from previous sessions. The EG couples participated in 10 small group sessions (6–8 couples assigned to each group). The group sessions involved work on individual feelings and affective bonds, with specific objectives for the man and the woman in each participating couple. The conceptual differences between this intervention and the usual programmes of maternal education are primarily the following:

1) a preparation for parenting and not just for the childbirth, which (although obviously an important event for parents) is considered as just the first step of the perinatal period;

2) an overall preparation for both parents and not only an intervention for women; and

3) the fundamental basis of the approach itself: an overall psychosomatic approach versus the antenatal psychoprophylaxis, with each person building his or her own model of the physical, emotional and social experience.

The weekly sessions began during the second term of pregnancy and lasted two hours and 15 minutes. The sessions were carried out at the end of the afternoon to facilitate participation by those who work. Each session consisted of an interactive exchange of information (60%) and practical exercises (40%). Between sessions, a follow-up phone call was included to avoid participant attrition and to record any unusual incident.

For quality control, intervention sessions were videotaped and reviewed in two ways: 1) a nurse facilitator reviewed videotapes and provided feedback to the first author; 2) the first author provided weekly individual supervision to the facilitators for each intervention group to ensure similar objectives to the course content.

In the control group (CG), participants were free to choose whether or not to participate in standard antenatal education programmes in accordance with the existing protocol at their centre of reference. These programmes offer eight sessions of two hours each during the third term of pregnancy; the focus is childbirth and pregnancy health. No information is included about body sensations or individual experience, neither for men nor women, and no follow-up phone calls are made. There are no open sessions without topic. Each group is open and can receive 12 couples or more (at least twice the size of the EG programme). Each session includes a time for giving information (75%) and a time of relaxation exercises (20%), with the other 5% for questions. The duration of the session is similar to the EG session; however, the schedule and content of the CG sessions prevented regular or frequent participation by men with a standard work schedule.

### Statistics

Quantitative data were analyzed with SPSS for Windows, version 15. All analyses were conducted on the basis of intention-to-treat. Summary statistics were compiled for the EG and CG couples on EPDS, social support, stress, couple relationship and postpartum variables (women’s somatic symptoms, childbirth outcomes and participation satisfaction of couples). Group differences in baseline characteristics were examined using chi-square tests, the Mann–Whitney test or the Kolmogorov-Smirnov test. Categorical data of principal variables are presented as percentages and comparisons made by chi-square tests. Univariate analysis used average and standard deviation by 95% confidence interval. Analysis of secondary variables applied the Student *t* test and the chi-square test.

## Results

A preliminary analysis was performed to compare the studied centres and no significant difference was obtained; the results from the three centres were comparable in all prenatal and postnatal variables.

### Participants

The intervention group included all future fathers and mothers assigned in EG. The women completed all the questionnaires, the men completed questionnaires only concerning relationship. Both signed the formal consent. Of the 220 couples initially selected to participate, 184 were included (CG = 92 and EG = 92) and 36 were excluded for the following reasons: miscarriage (n = 4), high-risk pregnancies with special medical protocol (n = 9), family violence (n = 5), decided to stop their participation (n = 6), and migrated to another country (n = 12). Between T_1_ and T_2_, there were 37 attritions: 13 explained and 24 unexplained. Due to causes beyond control, 21 envelopes with completed questionnaires were lost in the mail. In addition, seven questionnaires were rejected because they were not completed correctly. Of 147 reportedly completed questionnaires, 127 were analyzed (Figure 1). Global losses were higher from the CG than the EG: 36% (34) versus 25% (23) respectively.

### Socio-demographic characteristics

The study groups were comparable on all prenatal variables. The average age was 29.30 years (28.36 to 30.25: 5.53 SD); 11 women were aged 18 to 20, and two were over 40 (42 and 43 years). The education profile was 14% primary education, 29% with secondary education, 14.90% with initial professional training, 16.40% with completed professional training, 14% indicated access to college, and finally, 11.70% had university education. Socioeconomic status was 14.13% from the middle class, 24.73% between working and lower class, 34.86% from the lower class and 26.28% below the poverty threshold. The variable “pregnancies” indicated that 22.30% of participants had a miscarriage or abortion history, with an average of two (1.3 SD); five women had more than three previous miscarriages. The variable Psychological treatment before pregnancy indicated that 29.19% of participants were treating for various reasons. Finally, the average antenatal risk of PPD evaluated with interview was 4 (1.8 SD) which represents a moderate risk (Table 3).

**Table 3:** Demographic and obstetric variables for control participants versus intervention participants

**Variables**	**Groups**		**P**
	**Control**$\bar{x}$ **(SD)**	**Intervention**$\bar{x}$ **(SD)**	
Age, years	28.5 (6.2)	29 (5.2)	0.21*
Previous pregnancies number	2.05 (1.2)	2.01 (1.2)	0.35*
Antenatal risk of PPD (interview)	4.12 (1.8)	4.47 (1.9)	0.14*
	%	%	
First pregnancy	62.00	65.20	
Multiparous	38.00	34.80	0.38**
**Origin**			
Spanish	59.08	54.30	
Another European country	3.30	3.30	
Non-European country	37.00	42.40	0.74**
**Socioeconomic level** (mean annual)			
Poverty: ≤ 10000 $ USA	28.18	26.16	
Working class: 18400 to 20000 $	35.86	30.04	
Low-middle class layer: 22000 $	21.73	30.02	0.40**
Middle class: 24000 to 27400 $	14.13	13.04	
Psych treatment before pregnancy	27.49	32.09	0.41**
Health problems before pregnancy	20.70	20.03	0.85**

*P evaluated with *t* de Student.

**P evaluated with Chi-Square test Pearson.

### Postpartum depressive symptoms

The assessment of PPD risk, defined at the cut-off point ≥12 on the EPDS scale and realised between the 5^th^ and 12^th^ week following childbirth (9.35 weeks, 3.56 SD), identified 39.34% (51) of women at risk of PPD. The rate obtained is high, confirming that the sample of participants presented a moderate to high risk: 45.50% (27) of women in the CG and 34.30% (24) in the EG; the 11.20% difference between the two groups was not significant (p = 0.26). These results are shown in Tables 4 and 5.

**Table 4:** **Clinical data comparing control participants versus intervention participants in T**_**2**_

**Postpartum variables**	**Groups**		**P**
	**Control**$\bar{x}$ **(SD)**	**Intervention**$\bar{x}$ **(SD)**	
Depressive symptoms (score EPDS)	11.11 (6.05)	9.34 (5.18)	0.08*
			0.12**
Stress	203.29 (114.96)	190.10 (123.48)	0.56*
			0.42**
Social support	29.02 (9.08)	27.41 (8.32)	0.34*
			0.35**
Women dyadic adjustment	103.60 (28.99)	108.98 (24.61))	0.30*
Couple relationships (DASS)			0.39**
Men dyadic adjustment	124.80 (18.89)	129.10 (10.95)	0.32*
Couple relationships (DASS)			0.53**
Birth weight	3019,01 (668,83)	3301,87 (506,65)	0.01*
	% (n)	% (n)	
Incidence preterm birth	22,4 (13)	4,4 (3)	0.003***
Risk of PPD (EPDS ≥12)	45,5 (27)	34,3 (24)	0.26***

*t Student test.

**MannWhitney U test.

***Chi-Square test Pearson.

**Table 5:** Antenatal and postpartum clinical data

**Variables**	**Control G**$\bar{x}$ **(SD)**				**Intervention G**$\bar{x}$ **(SD)**	
	**Antenatal (SD)**	**Postpartum (SD)**	**P**	**Antenatal (SD)**	**Postpartum (SD)**	**P**
Depressive symptoms (EPDS)	10 (5.84)	11.11 (6.05)	0.36*	11.23 (5.75)	9.34 (5.18)	0.01*
			0.23**			0.01**
Stress no events	4.58 (2.76)	5.50 (2.38)	0.72*	5.41 (3.32)	4.88 (2.61)	0.21*
			0.53**			0.40**
Stress score value	189.68 (114.65)	203.29 (114.96)	0.58*	212.09 (131.41)	190.10 (123.48)	0.19*
			0.67**			0.27**
Lack of social support	26.59 (8.06)	29.02 (9.084)	0.26*	26.81 (8.25)	27.41 (8.32)	0.92*
			0.18**			0.94**
Global DASS women	116.37 (24.46)	103.6 (28.99)	0.008*	119.91 (25.97)	108.98 (24.61)	0.0001*
			0.002**			0.0001**
Global DASS men	125.81 (14.28)	124.80 (18.89)	0.23*	122.68 (17.85)	129. 10 (10.95)	0.69*
			0.13**			0.5 3**

*t student test.

**Wilcoxon test.

The average postnatal EPDS differed 1.76 ($\bar{x}$ = 11.10, 6.05 SD, in CG; $\bar{x}$ = 9.34, 5.17 SD, in EG); although not significant, this difference represents a trend (p = 0.08). No changes were detected between the prenatal and postnatal EPDS results for women in the CG (P = 0.36). By contrast, women in the EG had a significant postnatal decrease in the number of depressive symptoms (P = 0.01) when compared with the prenatal test (Table 5). More details of these analyses were included in an article published earlier, which focused on two results: 1) the relationship between PPD risk and preterm birth in all participants, and 2) 59.70% of women in the experimental group decreased their depressive symptoms comparative to the antenatal assessment [56].

### Premature childbirth and birth weight

The rate of premature births differed significantly between groups: 22.40% (13) in the CG and 4.40% (3) in the EG; the chi-square test showed a significant difference (p = 0.003); six babies were born before the 33rd week of gestation (29–33), five in the CG and one in the EG (Table 4).

The CG babies weighed significantly less (by an average of about 300 g) than EG babies, 3019.01 g (SD = 668.83) versus 3301.87 g (SD = 506.65) respectively. The difference was statistically significant when calculated using the Student *t* test (p = 0.01), which is presented in Table 4.

### Analysis of social support, stress and, couple relationship variables

The analysis of social support variable reveals a deficit of social support in all cases. The stress variable analysis indicates great stress-causing events in all women with a number of 4.5 - 5 stress events. The average of after-birth social support and stress events did not change when comparing the antenatal evaluation and no difference was observed between groups (Table 5). There was no change in the “relationship with partner” variable in men after childbirth, but it decreased significantly in women who indicated relationship loss or a lack of relationship adjustment after childbirth; the difference was more significant in EG participants (Table 5).

On account of the size of the final sample, we do not have made separate analyzes between each of the psychosocial variables and the premature childbirth.

### Satisfaction analysis of the antenatal programme

We evaluated the satisfaction of the antenatal programme intervention received by all participants. To do this, we used only the affirmative responses of participants (“yes, it helped me”; “yes, I'm satisfied”). The percentage of fully completed satisfaction questionnaire from CG participants was 15% (only 25% participated more than seven sessions in a standard intervention) and from the EG was 85.70% (88% participated more than seven of the ten sessions); these comparative analyses were evaluated with the chi square test. The results obtained are as follows:

1) There is a significant difference between the groups for questions a, b, c, d, e

a. understanding the symptoms of pregnancy: 4 in the CG and 38 in the EG (p = 0.05)

b. understanding all information received: 3 in the CG and 37 in the EG (p = 0.02)

c. understanding attitudes towards delivery, through video: 3 in the CG and 38 in the EG (p = 0.05)

d. expressing my feelings: 0 in the CG and 18 in the EG (p = 0.04)

e. communicating with the baby: 3 in the CG and 38 in the EG (p = 0.05)

2) No difference is noted for other questions: clarifying various information about pregnancy (4 in the CG and 27 in the EG: p = 0.45); changing erroneous beliefs (4 in the CG and 31 in the EG: p = 0.35); reducing my loneliness (4 in the CG and 30 in the EG: p = 0.37); couple communication (3 in the CG and 21 in the EG: p = 0.70); improving support in the couple (1 in the CG and 21 in the EG: p = 0.10); requesting overall support (0 in the CG and 9 in the EG: p = 0.16); I am satisfied with the intervention (12 in the CG and 48 in the EG: p = 0.28).

## Discussion

The high percentage of postpartum depressive symptoms (39.34% with EPDS score ≥12) among all participating women confirms that socioeconomic status has a high impact on PPD risk. It follows then that selecting only at-risk cases leads to a high number of postnatal depressive symptoms. The situation of risk-facing women with a more disadvantaged socioeconomic status deserves some consideration. This high rate (45.5% in the CG; 34.3% in the EG) suggests that it is very difficult to reduce the risk of PPD for these women with only 10 prenatal group sessions, and in this respect the aim of this study was very ambitious. This is a complex group of women necessitating a complex psychosomatic intervention during the antenatal period.

The initial sample in this study was also decreased, which limits the conclusions that can be drawn. More data were lost in the control group; this could be one reason for the difference between groups of variables. However, it is worth highlighting that the average EPDS was 1.68 points lower for women in the EG. A third assessment of EPDS would have been useful. Future studies should incorporate a longer follow-up to establish whether this reduction remains stable over time or undergoes a change.

Attrition is a frequent risk in longitudinal randomized pregnancy studies [57] and one of the limitations of PPD preventive studies [33]. In this study, more cases than the average were lost in the computer system or in the mail. Address changes, migration, domestic violence, and extreme poverty hampered the collection of postpartum data in general. It is possible that among the missing questionnaires were many couples who had separated, which could explain some results, such as those observed regarding the relationships in which women adjusted poorly compared to the men. Presumably, some of the men did not respond because they were away from home when the postpartum questionnaires were sent.

As the partner relationship has been associated with PPD in previous studies, this study measured this variable in both women and men to compare responses. Furthermore, the separation of socioeconomically disadvantaged couples is common after childbirth, and some of our participating couples in both groups separated or experienced relationship adjustment problems. Although no change was observed among the male respondents, the women reported a greater lack of adjustment in their relationship with the baby’s father. In our study, we were somewhat surprised to observe greater relationship loss among the EG participants. We hypothesize that the separation rate and other relationship problems were underreported in the CG, because these events cause many women in these situations to change their residence and thus they are lost to follow-up. Perhaps because of the stronger relationship with programme staff, fewer EG male and female participants were lost to follow-up and therefore more data were available on the relationship status in this group.

Notably, the study did not have any funding to compensate participants. The women in the EG expressed their satisfaction with the intervention and responded willingly to the questionnaires. The high level of adherence to the experimental intervention (88% of women participated in more than seven of the ten sessions) is another indicator of satisfaction. By contrast, the women in the CG who were depressed after giving birth and did not receive any support from the study were probably not likely to be motivated to complete the questionnaires.

It is worth highlighting that 43% of the participants in the present study were immigrants, most of them recently arrived in Spain or France. In a Vancouver study conducted with 594 women, recent immigration (within five years) was identified as a predictive variable for PPD; the other predictors were a history of depression, a vulnerable personality, stress and low perception of social support [58]. The immigrant experience tends to involve a low level of social support during the first years. In this study, a lack of social support is associated with postnatal depressive symptoms [56]; this is a very difficult problem to avoid with immigrant mothers. Even when help was provided through the intervention group, it was not enough. A US study using the Centre for Epidemiological Studies-Depression scale (CES-D ≥ 16) to assess immigrant Hispanic women (n = 3952) showed that most displayed depressive symptoms during their pregnancy and many (42.6%) continued to display symptoms of PPD. This depression was associated with a low education level and immigrant status [59].

The US study conducted by Boury et al. [60] within the *Special Supplemental Feeding Program for Women, Infants and Children* (WIC) indicated that 51% of the women evaluated using the BDI 30 weeks after childbirth, displayed symptoms of depression associated with stress and lack of social support. Rubertsson et al. [27] also found that stress influences the risk of PPD. Some of the women in the EG, who were immigrants with no stable social ties, did not show a more positive result; some cases were highly precarious. Their living conditions may have been so difficult that it was impossible to protect them from depressive symptoms with prenatal group intervention. Further complementary action would have been required, such as, for example, on-going postnatal support or working on their basic needs, as suggested by Polomeno [61].

While some studies have shown a decrease in symptoms of PPD [20,32,39-43,62-64], others have not [37-39,45,46,65-68]. Among the studies that show a positive impact, only four used a rigorous scientific methodology, but the sample was very small [42,43] or attrition was a limiting factor [33]. Although the risk of PPD did not decrease significantly in our study, other aspects of the experimental intervention were assessed. For example, awareness of what happens in the body of the mother can positively influence childbirth because she understands what is happening and this understanding is relaxing [30]. Body image satisfaction is an important psychological determinant of depressive symptoms in pregnancy [69], as improving body image is important. Still, when the mother is aware of her body and its sensations she can take better care of her health, which can result in a positive result in the birth of her baby. Future research about PPD prevention and childbirth outcomes is needed.

The premature births rates in our study were 22.4% (13) in the CG and 4.4% (3) in the EG (p = 0.003), similar to those obtained by Field et al. [20] with 16.4% premature childbirth in the control group and 4% in the massage intervention group (p = 0.004). These results deserve at least a reflection on the advantages of considering an integrated approach to body and mind for pregnant women at risk of PPD. Pregnant women with depressive symptoms tend to have low self-esteem and poorer body image [69], these aspects can be improved with working on body awareness [30]. This study could not analyze as many variables as desired, but we note that body consciousness in pregnant women is inseparable from the consciousness of the baby in utero and this connection has repercussions. This study cannot speak of any association between body awareness and birth outcome, but in a previous study we analyzed depressive symptoms and prematurity and, we found that there is a relationship [56]. We have not studied the weight gain during pregnancy and this is a limit to our results since this variable might be associated with prematurity. However, we did not obtain differences between groups regarding the other variables that influence prematurity (complications during pregnancy and abortions before pregnancy).

This study underlines the importance of identify women at risk of PPD, provide interventions to prevent PPD in vulnerable women and, protect their babies’ from premature birth. All this was recommended by authors who have observed that PPD was associated with earlier gestational age and lower birth weight [70]. Our study was designed with this purpose and results support what has been observed by other authors.

If resources are available, it is advisable to administer the questionnaires in face-to-face interviews in order to avoid their loss or response errors. It is important to add to this limitation the number of cases studied (a larger sample is advisable) and the evaluation of depressive symptoms at only two stages (a third evaluation might show how the symptoms would have evolved). Lastly, the absence of a clinical interview is another limitation; this could have been remedied by adding another questionnaire as a comparison to the EPDS. We think that these limits prevent seeing the variable PPD as a mediator of premature childbirth, could be an underestimation of the participants in two postpartum questionnaires, the social support and the EPDS.

## Conclusions

The aim of the study was to evaluate the impact of a novel psychosomatic antenatal programme meant to decrease symptoms of depression (primary outcome) and preterm birth (secondary outcome). This study was quite ambitious in its context of working with women at psychosocial and socioeconomic risk (moderate to high levels). The experimental intervention using a psychosomatic approach had an impact, but did not significantly lower PPD risk (due perhaps to both intrinsic and extrinsic factors). Nonetheless, the psychosomatic approach or another multidisciplinary approach is an option to consider in the antenatal care of vulnerable women in efforts to prevent PPD and premature childbirth. Both variables are important, and other studies suggest that levels of depression during pregnancy may contribute to other biomedical risk factors such as adverse obstetric, foetal, and/or neonatal outcome [71]; for this reason, interventions to decrease antenatal depressive symptoms are needed. Our study results demonstrate prevention possibilities and suggest interesting hypotheses for future interventions.

Preventing PPD requires that several aspects be taken into consideration: **1) early detection of PPD risk during pregnancy is feasible and incorporating it into a regular medical visit, such as echography, easily identifies women at risk; 2) preventive intervention during the antenatal stage is feasible with women at psychosocial risk,** but requires additional individual intervention in certain cases and/or subsequent postnatal intervention in many others. Antenatal programme intervention is necessary but not sufficient in preventing PPD; **3) A psychosomatic approach is a feasible preventive PPD intervention, but further studies are necessary to validate its efficacy; and 4) clinical interventions to identify and prevent PPD in vulnerable populations are important in the prevention of premature childbirth.**

### Recommendations

• Early detection of PPD risk during pregnancy, incorporating it into a regular medical visit such as echography, is recommended in PPD prevention.

• Preventive intervention during the antenatal period is feasible and adequate with women at psychosocial risk.

• Preventing PPD in vulnerable populations is important in the prevention of premature childbirth; we recommend these considerations all together.

## Competing interests

The authors declare that they have no competing interests.

## Authors’ contributions

MAO made principal contribution to conception and design, acquisition of data and ensuring that questions related to the accuracy of any part of the work are appropriately investigated. MAO and MH involved in drafting the manuscript and revising it critically for important intellectual content equally to this work. MS and JF participated in the design of the study and made substantial contributions to analysis and interpretation of data. All authors participated sufficiently in the work for appropriate portions of the content and given final approval of the manuscript to be published. All authors made substantial contributions to conception and design and given final approval of the version to be published.

## Authors’ informations

Maria Assumpta Ortiz Collado and Marie Hatem involved in drafting the manuscript and revising it critically for important intellectual content equally to this work.

## Pre-publication history

The pre-publication history for this paper can be accessed here:

http://www.biomedcentral.com/1471-2393/14/22/prepub
